# miR-181b regulates vascular stiffness age dependently in part by regulating TGF-β signaling

**DOI:** 10.1371/journal.pone.0174108

**Published:** 2017-03-21

**Authors:** Daijiro Hori, Brittany Dunkerly-Eyring, Yohei Nomura, Debjit Biswas, Jochen Steppan, Jorge Henao-Mejia, Hideo Adachi, Lakshmi Santhanam, Dan E. Berkowitz, Charles Steenbergen, Richard A. Flavell, Samarjit Das

**Affiliations:** 1 Department of Surgery, Johns Hopkins School of Medicine, Baltimore, Maryland, United States of America; 2 Department of Anesthesiology & Critical Care Medicine, Johns Hopkins School of Medicine, Baltimore, Maryland, United States of America; 3 Department of Cardiovascular Surgery, Saitama Medical Center, Jichi Medical University, Saitama, Japan; 4 Department of Cardiovascular Pathology, Ross Research Building, Johns Hopkins University, Baltimore, Maryland, United States of America; 5 Department of Immunobiology, Yale University School of Medicine, New Haven, Connecticut, United States of America; University of Southampton, UNITED KINGDOM

## Abstract

**Background:**

Endothelial dysfunction and arterial stiffening play major roles in cardiovascular diseases. The critical role for the miR-181 family in vascular inflammation has been documented. Here we tested whether the miR-181 family can influence the pathogenesis of hypertension and vascular stiffening.

**Methods and results:**

qPCR data showed a significant decrease in miR-181b expression in the aorta of the older mice. Eight miR-181a1/b1^-/-^ mice and wild types (C57BL6J:WT) were followed weekly for pulse wave velocity (PWV) and blood pressure measurements. After 20 weeks, the mice were tested for endothelial function and aortic modulus. There was a progressive increase in PWV and higher systolic blood pressure in miR-181a1/b1^-/-^ mice compared with WTs. At 21 weeks, aortic modulus was significantly greater in the miR-181a1/b1^-/-^ group, and serum TGF-β was found to be elevated at this time. A luciferase reporter assay confirmed miR-181b targets TGF-βi (TGF-β induced) in the aortic VSMCs. In contrast, wire myography revealed unaltered endothelial function along with higher nitric oxide production in the miR-181a1/b1^-/-^ group. Cultured VECs and VSMCs from the mouse aorta showed more secreted TGF-β in VSMCs of the miR-181a1/b1^-/-^ group; whereas, no change was observed from VECs. Circulating levels of angiotensin II were similar in both groups. Treatment with losartan (0.6 g/L) prevented the increase in PWV, blood pressure, and vascular stiffness in miR-181a1/b1^-/-^ mice. Immunohistochemistry and western blot for p-SMAD2/3 validated the inhibitory effect of losartan on TGF-β signaling in miR-181a1/b1^-/-^ mice.

**Conclusions:**

Decreased miR-181b with aging plays a critical role in ECM remodeling by removing the brake on the TGF-β, pSMAD2/3 pathway.

## Introduction

Hypertension is a major cause of morbidity and mortality worldwide. Increased aortic stiffness is one of the major factors that disproportionately increases central arterial pressure, creates systolic hypertension, and increases pulse pressure.[1–4] The remodeling of extracellular matrix (ECM) by decreased elastin or it’s fracturing, increased collagen deposition, endothelial dysfunction, and cross-linking of ECM, are the major contributors of increased vascular stiffness.[1, 5]

Many miRNAs contribute to vascular dysfunction, but among these, miR-145 and miR-181b have been found to be key regulators of vascular inflammation in ApoE signaling pathways.[6, 7] In the ApoE-deficient mouse model, delivering miR-181b was found to protect against vascular inflammation by directly binding at the 3'-UTR of importin-α3, a key regulator of the NF-κB signaling pathway.[7, 8] From human plasma samples, it has been shown that in coronary artery disease, the circulating level of miR-181b expression is significantly lower compared to healthy controls.[7] Furthermore, miR-181a has been reported to be decreased in vascular smooth muscle cells (VSMCs) when stimulated by angiotensin II, whereas overexpression of miR-181a resulted in inhibition of adhesion of VSMCs to collagen and reduced expression of osteopontin, a multifunctional protein found in abundance in atherosclerotic plaques.[9] The miR-181 family has also been reported to play an essential role in early NKT cell development by targeting 3'-UTR of PTEN in the thymus.[10] Together, these results suggest that the miR-181 family is an important modulator of vascular inflammation.[11] However, the role of the miR-181 family in overall vascular function, including endothelial function and vascular stiffness, remains unknown.

We hypothesized that the chronic depletion of miR-181a/b will lead to vascular stiffening either by endothelial dysfunction or by ECM remodeling or their combination. To study this, we used miR-181a1/b1^-/-^ mice, and compared their aortic stiffness and blood pressure with WT mice. Better understanding of the role of miR-181 in the development of vascular stiffness and systolic hypertension may lead to novel therapeutic approaches in the future.

## Material and methods

### Animals

**Fig 1:**
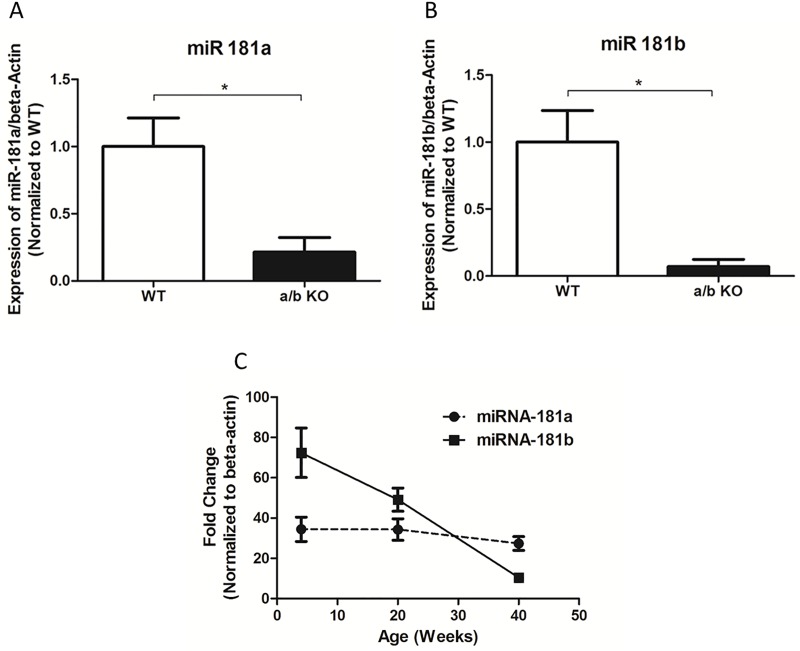
**A, B, qPCR of miR-181a and miR-181b in the aortic tissue.** The minimal expression of miR 181a (A) and miR 181b (B) in the aortic tissue represents cross talk between miR-181a primer vs miR-181c primer, and miR-181b primer vs miR-181d primer. This is due to 99.9% homology between the sequences (miR-181a and miR-181c; miR-181b and miR-181d). We used Qiagen’s SYBR platform, and it has been documented that there is ~10% cross-reactivity among the same family member miR-RNAs.[13] **C**, Relative expression of miR-181a and miR-181b at 4 weeks, 20 weeks and 40 weeks of age in WT mice aorta (n = 3–4). Values are mean ±SEM *p<0.05, **p<0.01, ***p<0.001.

We used male mice deficient for the miRNA clusters miR-181a1b1 (containing the miR-181a-1 and miR-181b-1 cluster, located on chromosome 1) (miR-181a1/b1^-/-^).[10] qPCR data confirmed that both miR-181a1 and miR-181b1 are knocked-out from the aortic tissue of the mice (Fig 1A and 1B). This mouse model was generated in the C57BL6/J genetic background.[10] Chimeric offspring were backcrossed up to ten generations with C57BL/6 mice. Germline transmission was confirmed by PCR of tail genomic DNA with the primers 5′-AAATGCTTATTCCATGCACATT-3′ and 5′- ATCAACGGTCGATGGTTTT-3′ which amplifies 554 and 647-base pairs products from the wildtype allele and floxed allele, respectively. The primers 5′- AAATGCTTATTCCATGCACATT-3′ and 5′-TGAGCCCCTGGATAACAAAG-3′ amplify a 377-base pair product from the deleted allele. Consistent with the previous study using the same genotyped mice [10, 12], we also used C57BL/6 mice as the control group. All mice had the same C57BL/6 background. 25 mice from both phenotypes were used for these experiments. They were provided with food and water ad libitum, and house them in 7 am to 9 pm light and rest of the time dark cycle facility. Mice were treated humanely and all experimental procedures were approved by the Institutional Animal Care and Use Committee of Johns Hopkins University. Animal care staffs regularly monitor the mice, and all the animals looked healthy during the experimental protocols. We used Ketamine-Xylazine to anesthetize the animal (check for total anesthesia by toe pinching) before we excise the organs.

### Non-invasive Pulse Wave Velocity (PWV) Measurements

A high-frequency, high-resolution Doppler spectrum analyzer (DSPW, Indus Instruments, Houston, TX, USA) was used. Mice were anesthetized with 1.5% isoflurane, and were placed in a supine position on a heated pad equipped with EKG. The animals were allowed to stabilize to a physiologic heart rate following which a 20 MHz probe was used to measure the descending aortic and abdominal aortic flow velocities. The time between the R wave of EKG to the start of pulse wave form for each measurement location was calculated using a real-time signal acquisition and spectrum analyzer system.[14, 15]

### Non-invasive blood pressure measurement

Blood pressures (BP) were measured by tail-cuff non-invasive BP measurement system using volume pressure recording sensors (CODA, Kent Scientific Corp, Torrington, CT, USA). The animals were trained in a holder every day for 1 week. BP measurements were performed on a heated platform set at 37°C. Occlusion cuff and volume pressure recording cuff was placed on the tail and 20 measurements were acquired. The average of at least five consecutive readings were used for the analysis.[16]

### Tensile testing

Thoracic aortas were harvested and cut into 2mm rings. The aortic ring and a 0.5mm segment proximal to each ring was imaged at ×10 magnification to measure inner diameters (D_i_), outer diameters (D_o_), and the vessel length (L). The diameter and the length were measured with Image J software (National Institutes of Health [NIH], Bethesda, MD, USA). The aortic rings were mounted onto the pins of an electromechanical puller (DMT560; Danish Myo Technology A/S, Aarhus, Denmark). After calibration and alignment, the pins were slowly moved apart using an electromotor at a rate of 20 μm/sec to apply radial stress on the specimen until breakage. Displacement and force were recorded continuously. Engineering stress (S) was calculated by normalizing force (F) to the initial stress‐free area of the specimen (S = F/2t×l; where t = thickness and l = length of the sample). Engineering strain (λ) was calculated as the ratio of displacement to the initial stress‐free diameter (Di). The stress‐strain relationship was represented by the equation S = α exp (βλ), where α and β are constants. α and β were determined by nonlinear regression for each sample using Excel (Microsoft, Redmond, WA, USA).[5]

### Decellularization

Aortic rings were harvested from 21 week old miR-181a1/b1^-/-^ mice and C57BL6 mice following a protocol described previously[15] with minor modifications. A 0.1% SDS solution was prepared in 50mM NH4OH. The aortic rings were placed in the microcentrifuge tubes containing the solution and were placed on the vortexer/shaker for 24 hrs following which the rings were rinsed with PBS for 15 minutes on the vortexer/shaker twice.

### Endothelial function (vasoreactivity)

Thoracic aortas were isolated, and trimmed into 2mm rings and mounted on a wire myograph (DMT). One end of the aortic ring was connected to a transducer and the other to a micromanipulator. The vessels were immersed in a bath filled with constantly oxygenated Krebs buffer (NaCl 118.3 mM, KCL 4.7 mM, CaCl2 1.6 mM, KH2PO4 1.2mM, NaHCO3 25mM, MgSO4 1.2mM, and Dextrose 11.1mM) at 37°C. The vessels were stretched to an optimal resting tension using the micromanipulator. 60 mM KCl was administered, and repeated after three washes with Krebs buffer. Vessels were then allowed to equilibrate for 1 hour. The vessels were pre-constricted with Phenylephrine (1 μM) for 15 minutes and ACh-induced dose response relaxations (1nM to 10uM) and SNP-induced dose response relaxations (1nM to 10uM) were measured. Relaxation responses were calculated as a percentage of tension after preconstriction.[14, 17, 18]

### No production measurement

Excised aortas were cut open to make aortic strips 2-4mm in length. The aortic strips were pinned, endothelial side up in a Silastic-coated Petri dish containing Krebs-HEPES buffer (in mM): 110 NaCl, 4.7 KCl, 25 NaHCO_3_, 1.2 MgSO_4_, 1.03 KH_2_PO_4_, 11.1 d-(+)-glucose, 20.0 HEPES, and 1.87 CaCl_2_, pH 7.4 at 24°C. Tissues were incubated with 1 nM of the NO-sensitive fluorescent dye 4-amino-5methylamino-2′,7′-diaminofluoroscein (DAF-FM) diacetate (Molecular Probes, Eugene, OR, USA) for 30 min at 37°C. DAF-FM was washed out followed by 20-min equilibration period. Fluorescence intensity data were collected using the Nikon NIS-Element suite (Nikon Corporation, Shinagawa, Tokyo, Japan), with excitation and emission wavelengths set to 485 and 510 nm, respectively. Fluorescence intensity was recorded every 30 s for a period of 10 min. Acetylcholine (10uM) was administered on to the sample and the slope of intensity change was calculated for a period of 5 minutes to indicate rate of NO production. Data was then normalized to WT baseline measurement = 100%.[19]

### TGF-β and angeotensin II measurement

TGF-β1 ELISA kit (R&D Systems Inc., Minneapolis, MN, USA) and Angiotensin II EIA kit (Sigma-Aldrich, St. Louis, MO, USA) were used to detect TGF-β and Angiotensin II from mice serum and cell culture media as directed by the manufacturer’s instructions.

### Cell culture

Aortic rings were harvested from 10 week old miR-181a1/b1^-/-^ mice and C57BL6 mice following a protocol described by Kobayashi et al.[20] with minor modifications. The rings were cut open to make aortic strips and then were placed into microcentrifuge tubes containing 2 mg/ml collagenase. The tubes were gently rocked for 30 minutes at 37°C before centrifugation at 3000 rpm for 10 minutes. Supernatant was discarded and the pellets were resuspended in DMEM containing 20% serum and transferred into 30 mm cell-culture dishes. After 2 hours, non-adhered debris was removed and replaced with ECM media (ScienCell Research Laboratories, Carlsbad, CA, USA) for endothelial cell culture. The media were replaced every 48 hours until VECs reached confluence. Aortic strips were further transferred to 30 mm dishes containing DMEM supplemented with 20% serum for VSMC culture. Samples were maintained for 7–10 days to obtain confluent VSMCs. At passage 2, media was washed out and were replaced with serum free media. After 24 hours of incubation, media was collected, centrifuged and the supernatant was stored at -80°C for TGF-β measurement by ELISA. Cells were lysed into 1xRadioimmunoprecipitation assay (RIPA) buffer containing protease and phosphatase inhibitors.

### Immunoblot analysis

30 μg of protein were resolved by SDS-PAGE and electro-transferred to PVDF membranes for western blotting. Primary antibodies that recognize p-SMAD2/3 and SMAD2/3 were from Santa Cruz Biotechnology (Rabbit). The p-eNOS (Ser 1177) and eNOS antibodies were from Cell Signaling Technology (Danvers, MA, USA). The α-tubulin and Angiotensin II receptor 2 antibodies were from Abcam (Cambridge, MA, USA). VE-Cadherin and α-Actin were from Santa Cruz Biotechnology (Starr County, TX, USA). Immunoreactive proteins were visualized using an enhanced chemiluminescence analysis kit (EMD Millipore, Billerica, MA, USA).

### Immunohistochemical (IHC) staining

Mice aortic tissue were fixed in formalin, and embedded in paraffin as described before.[5] p-SMAD2/3 (Cell Signaling Technology, Danvers, MA, USA), Masson-Trichrome, and H&E were performed on 5-μm transverse sections of the aorta.

### Digital image analysis

Slides that were stained for collagen and pSMAD2/3 were digitized on an Aperio AT system (Leica Biosystems, Nussloch, Germany). Using Aperio ImageScope tools, the area of staining were measured. That area was divided by the total area of the vascular layer or the vascular media in the same aortic ring section.

### Luciferase reporter assays

1.25 X 10^6^ rat aortic vascular smooth muscle cells (A7r5) were transfected with 50 ng of TGF-βi 3'-UTR reporter construct (GeneCopoeia, Rockville, MD, USA) or with mutated TGF-βi reporter construct (mutagenesis at positions 72,74,76 and 77 from “A to C”, “A to C”, “G to C”, and “U to A”, respectively) with dual luciferase, firefly and renilla, which were then co-transfected with either scramble sequence or by mature miR-181b (GE-Dharmacon, Lafayette, CO, USA), using an electroporator (Neon Transfection System, Thermo Fisher, Carlsbad, CA, USA) following 1475 mV for 20 ms and 2 pulses. 48 hours after transfection, cells were washed lysed and assayed for firefly and renilla (served as internal control) luciferase activity using the Dual-Luciferase Reporter Assay System (Promega, Madison, WI, USA). The primer sequences to performed mutagenesis experiment are provided in Table 1.

**Table 1:** TGF-β1 and Mut TGF- β1 3'-UTR Primers.

	FWD Primer (5' - 3')	Rev Primer (5' - 3')
TGF-β1	AAAAAACTAGTCACCTCACTGGCTTTCGTG	AAAAAAAGCTTGGGTTAACAACCACGTTTGG
Mut TGF-β1	CAGTTTGCCAAAGAGACCAGTGACTGTTTTTGAAACCAAAGAGC	GCTCTTTGGTTTCAAAAACAGTCACTGGTCTCTTTGGCAAACTG

### Losartan preparation protocol

Losartan concentration of 0.6 grams/liter was prepared to give an estimated losartan dose of 50–70 mg/kg/day in the drinking water.[21, 22] 650 mg of losartan tablets were dissolved in 1080 ml of water to achieve a concentration of 0.6 g/L. The mixture was then double-filtered using a vacuum filtration system to remove the remaining excess sediment.[23] For cell culture purposes, we used 10 μM Losartan (Cayman Chemicals, Ann Arbor, Michigan, USA) for 48 hours.[24, 25]

### Statistical analysis

The results are presented as mean and standard error of measurement (mean ± SEM). Vascular relaxation for myograph was analyzed by t-test and LogEC50 by Extra sum-of-squares F test. For multiple comparisons, one-way analysis of variance (ANOVA) and the Bonferroni post hoc test were used. P<0.05 was considered as statistically significant. All analysis was performed using Prism 5 (GraphPad Software Inc., La Jolla, CA, USA).

## Results

### miR-181b but not miR-181a decreases with age

We have validated the miR-181a and miR-181b expression in the aorta of the miR-181a1/b1^-/-^ (a/b KO) mouse model using qPCR (Fig 1A and 1B). To see the temporal changes in miR-181a and miR-181b expression, WT mice aorta at different ages (4 weeks, 20 weeks, 40 weeks) were tested for miR-181a and miR-181b by qPCR. There was a decrease in miR-181b expression with increase in age (4 weeks: 72.39± 12.30, 20 weeks: 49.10± 5.75, 40 weeks 10.42±1.86, p<0.01). (Fig 1C) However, there were no significant changes in miR-181a expression in the aorta at different ages (4 weeks: 34.42±6.01, 20 weeks: 34.27±5.31, 40 weeks 27.38±3.40, p = 0.61). (Fig 1C)

### miR-181a/b regulates vascular stiffness and systolic blood pressure with age

**Fig 2:**
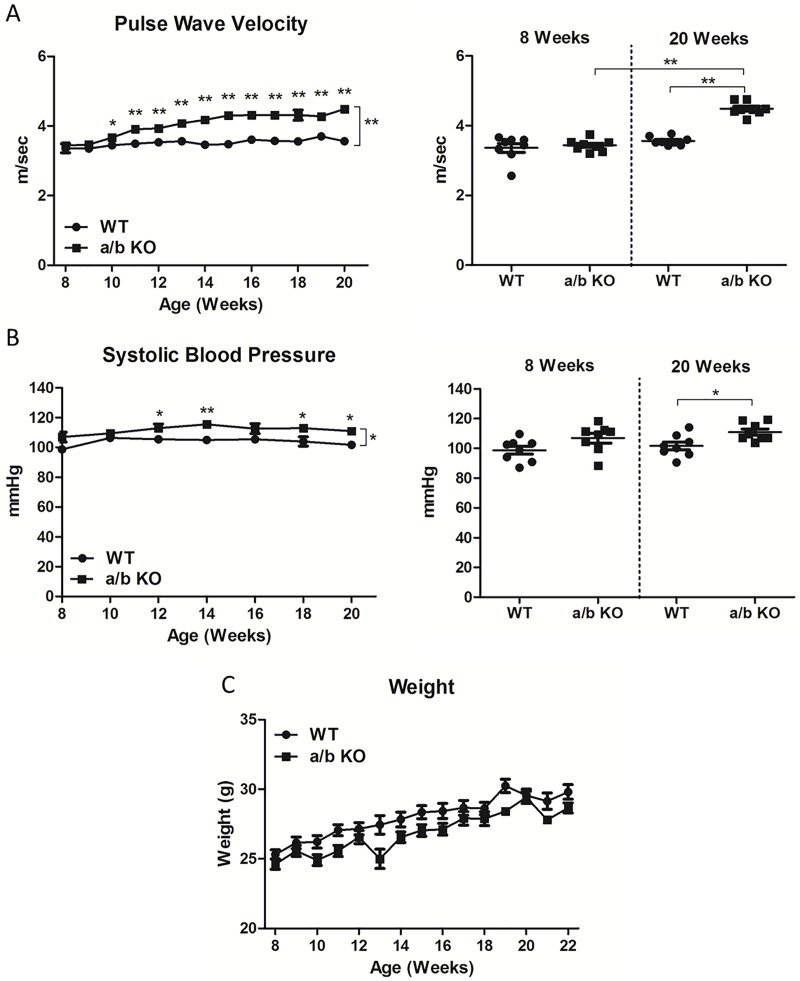
**A, Weekly pulse wave velocity and summary of pulse wave velocity at base line (8 weeks) and at 20 weeks of age comparing miR-181a1/b1^-/-^ (a/b KO) and control (WT) (n = 8) mice.** Significant difference was observed at 10 weeks. **B,** Bi-weekly systolic blood pressure and summary of systolic blood pressure at base line (8 weeks) and at 20 weeks of age between miR-181a1/b1^-/-^ (a/b KO) and control (WT) (n = 8). **C**, Body weight trends of both miR-181a1/b1^-/-^ and WTs with age. The increases in body weight were similar in both phenotypes, with trend towards less body weight in the miR-181a1/b1^-/-^. Values are mean ±SEM *p<0.05, **p<0.01, ***p<0.001.

PWV was measured weekly (n = 8) starting at 8 weeks of age for 12 weeks. The miR-181a1/b1^-/-^ (a/b KO) mice (Fig 2A) had an accelerated rate of increase in PWV compared to WT mice. Significant differences in the miR-181a1/b1^-/-^ (a/b KO) mice appeared as early as 10-weeks of age (n = 8) compared to the aged matched WT animals (WT: 3.450±0.034 m/sec, vs. a/b KO: 3.670±0.097 m/sec, p = 0.049). At 20 weeks, the difference was strikingly significant (20wk WT: 3.562±0.043 m/sec vs. a/b KO 4.489±0.068 m/sec, p<0.0001) (Fig 2A). Blood pressure was measured bi-weekly (n = 8), and higher systolic blood pressure in miR-181a1/b1^-/-^ mice (n = 8) compared to WT were observed (20wk WT: 102±2.6mmHg vs. a/b KO: 111±2.1mmHg, p = 0.015). (Fig 2B) On the other hand, there was no significant difference in diastolic blood pressure between the two phenotypes at 20 weeks (WT: 75 ± 2.9 mmHg vs. a/b KO: 80 ± 2.4 mmHg, p = 0.20). To ensure that the changes described in the current study are attributed to a miR-181a1/b1^-/-^ deficiency and not through an influence of body weight, body weight was checked weekly which showed similar changes in both phenotypes. (Fig 2C)

### Mechanical and functional properties of the aorta in the miR-181a/b^-/-^ mice

**Fig 3:**
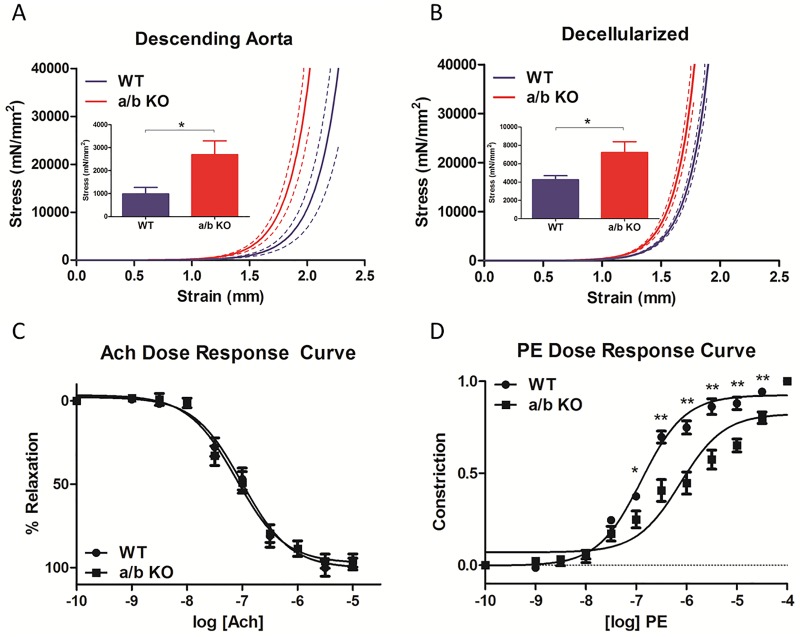
**A, Tensile testing of the descending aorta from miR-181a1/b1^-/-^ (a/b KO, n = 10) and control (WT, n = 11). B, Tensile testing of decellularized descending aorta from miR-181a1/b1^-/-^ (a/b KO, n = 9) and control (WT, n = 8)**. The solid line represent mean and the dotted line represents SEM. **C**, Dose-dependent response curve to acetylcholine (1nM– 10uM) in aortic rings from miR-181a1/b1^-/-^ (a/b KO, n = 14) and control (WT, n = 13), pre-constricted with phenylephrine (1uM). **D**, Phenylephrine dose response curve (1nM-10uM) of thoracic aorta from miR-181a1/b1^-/-^ (a/b KO, n = 12) and control (WT, n = 14). Values are mean ±SEM, *p<0.05, **p<0.01, ***p<0.001.

Tensile testing of the descending aorta performed at 21 weeks of age showed left ward shift in the stress- strain relationship of the miR-181a1/b1^-/-^ mice compared to WT in both intact rings and decellularized scaffolds. (Fig 3A and 3B) Endothelial dependent relaxation to acetylcholine after pre-constriction with phenylephrine were similar between the two phenotypes (WT: LogEC50–7.00±0.067, 95%CI -7.14 to -6.88 vs. a/b KO: -7.11±0.070, 95%CI -7.25 to -6.97). (Fig 3C) The response to phenylephrine was also tested by phenylephrine dose response curve. miR-181a1/b1^-/-^ mice were less responsive to PE compared to WT (WT: Log EC 50–6.90±0.045, 95%CI -6.99 to -6.81 vs. a/b KOs: Log EC 50–6.12±0.092, 95%CI -6.30 to -5.94). (Fig 3D)

### Endothelial NO production in the miR-181a/b^-/-^ mice

**Fig 4:**
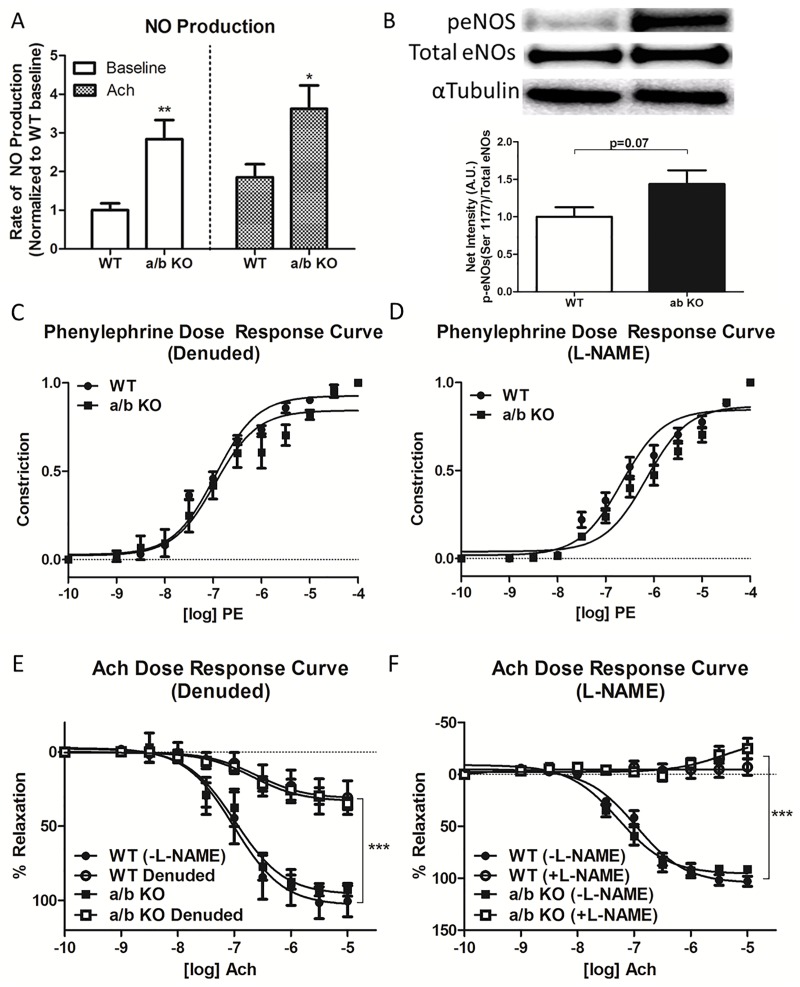
**A, Rate of nitric oxide (NO) production from the thoracic aorta of miR-181a1/b1^-/-^ (a/b KO) (n = 22) and control (WT) (n = 20).** Data are shown normalized to baseline WT aorta. **B**, Western blot of phosphorylated eNOS (p-eNOS) in the aorta of miR-181a1/b1^-/-^ (a/b KO) and control (WT) (p = 0.010, n = 6). **C**, Phenylephrine dose response (1nm-10uM) of the denuded thoracic aorta from miR-181a1/b1^-/-^ (a/b KO) and control (WT) (n = 4–5). **D**, Phenylephrine dose response (1nm-10uM) of the thoracic aorta treated with L-NAME (200nM) from miR-181a1/b1^-/-^ (a/b KO) and control (WT) (n = 5). Data are presented as % constriction from maximum constriction at 10uM. **E**, Dose response relaxation to acetylcholine (1nM-10uM) in denuded thoracic aorta and **F**, aorta pretreated with L-NAME (200nM) from miR-181a1/b1^-/-^ (a/b KO) and control (WT). The vessels were pre-constricted with phenylephrine (1uM). Values are mean ±SEM, *p<0.05, **p<0.01, ***p<0.001.

To determine if the differences in phenylephrine response curve was due to nitric oxide production, diaminofluorescein (DAF) fluorescence intensity was observed at baseline and after administration of acetylcholine in DAF loaded vascular strips. There was a significant increase in production of NO in aortic endothelium of miR-181a1/b1^-/-^ mice at baseline (WT: 1.0±0.18 vs. a/b KO: 2.8±0.49, p = 0.002) and following acetylcholine administration (WT: 1.9±0.34 vs. a/b KO: 3.6±0.61, p = 0.017) (Fig 4A). There was a trend towards increased p-eNOS at Ser 1177 in miR-181a1/b1^-/-^ compared to WT aorta (WT: 1.0±0.13 vs. a/b KO: 1.4±0.18, p = 0.07) **(**Fig 4B). To confirm that the shift in phenylephrine dose response was endothelium-dependent, phenylephrine dose responses were tested in endothelium denuded rings (WT: Log EC 50–7.03±0.05, 95%CI -7.13 to -6.93 vs. KOs: Log EC 50–6.83±0.13, 95%CI -7.09 to -6.57) (Fig 4C) and rings pretreated with the NOS inhibitor L-NAME (200 uM) (WT: Log EC 50–6.67±0.083, 95%CI -6.83 to -6.50 vs. KOs: Log EC 50–6.16±0.086, 95%CI -6.33 to -5.98). (Fig 4D) In both cases, the response of the miR-181a1/b1^-/-^ (a/b KO) was similar to WT (Fig 4C and 4D). Acetylcholine dose response curve was performed to confirm denudation and L-NAME treatment. (Fig 4E and 4F)

### Role of miR-181a/b in TGF-β-mediated pathways contributing to vascular stiffness

**Fig 5:**
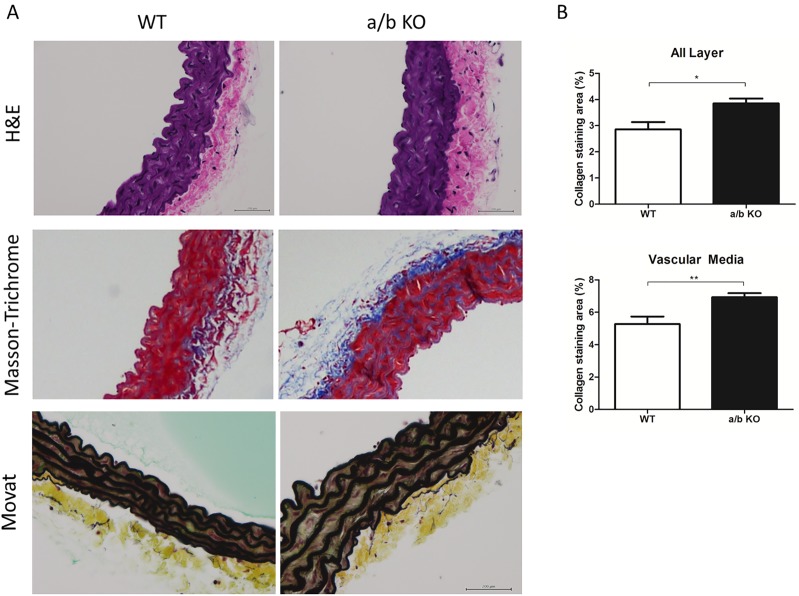
Histology of miR-181a1/b1^-/-^ (a/b KO) and control (WT). **A**, Top row shows H&E staining, followed by Masson-Trichrome staining and Movat’s staining. 20X magnification was used to take the pictures. **B**, Quantification of collagen staining for all layers and vascular media between miR-181a1/b1^-/-^ (a/b KO) and control (WT) (n = 5–6). Whole vascular ring were used for analysis. Values are mean ±SEM, *p<0.05, **p<0.01, ***p<0.001.

**Fig 6:**
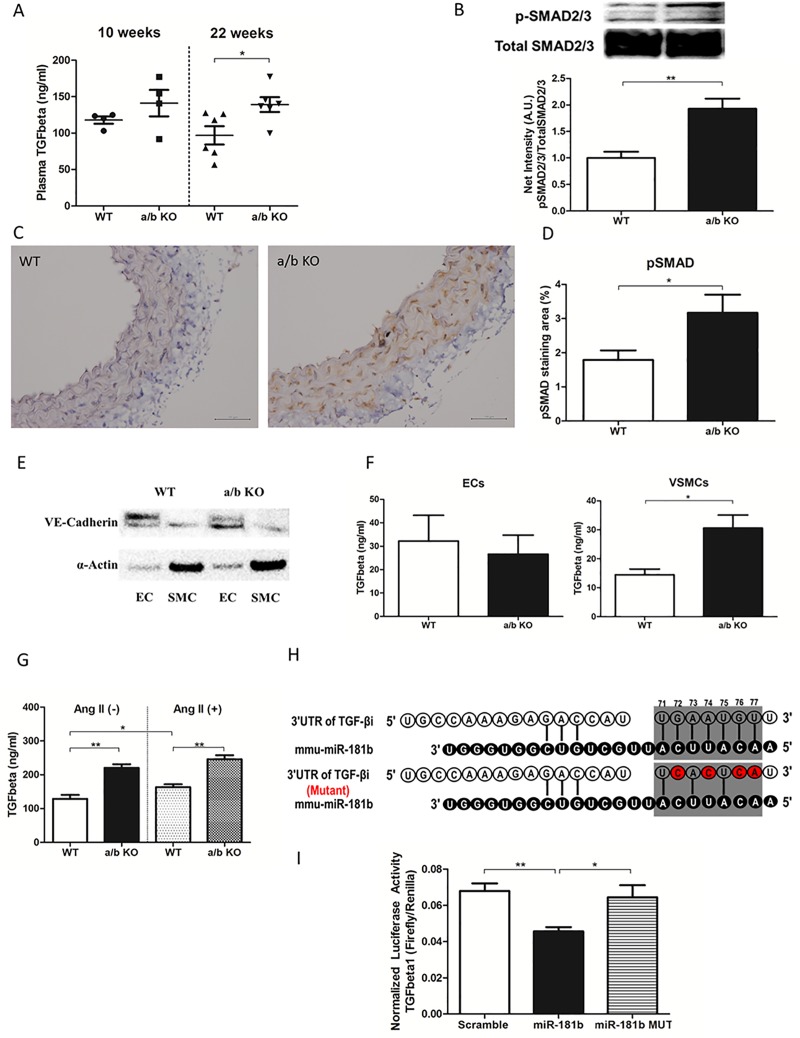
**A, Serum TGF-β of miR-181a1/b1^-/-^ (a/b KO) and control (WT) at 10 weeks of age (n = 4) and 22 weeks of age (n = 6). B, Western blot of p-SMAD2/3 compared to total SMAD2/3 in aorta of miR-181a1/b1^-/-^ (a/b KO, n = 4) and control (WT, n = 4) at 22 weeks. C, Immunohistochemistry showing pSMAD2/3 staining of the aorta from miR-181a1/b1^-/-^ (a/b KO) and control (WT). D**, Quantification of pSMAD2/3 staining for miR-181a1/b1^-/-^ (a/b KO) and control (WT). (n = 7) **E**, Westernblot showing VE-Cadherin (VEC marker) and α-actin (VSMC marker) of the cultured cells (n = 3). **F** TGF-β levels in the serum free media incubated for 24 hours in the VECs culture and VSMCs culture of miR-181a1/b1^-/-^ (a/b KO, n = 3) and control (WT, n = 3). **G**, TGF-β expressions in VSMCs of miR-181a1/b1^-/-^ (a/b KO) and control (WT), untreated and treated with angiotensin II. (n = 4–8) **H**, Using both TargetScan and DIANA-microT, we have predicted that position 71–77 of TGF-βi 3'-UTR can be a direct target of miR-181b (upper panel). We then performed mutagenesis at positions 72, 74, 76 and 77 from “A to C”, “A to C”, “G to C”, and “U to A”, respectively (lower panel). **I**, Luciferase activity of 3'-UTR of TGF-βi in rat aortic VSMCs (A7r5) co-transfected with TGF-βi or mutated TGF-βi reporter construct, along with miR-181b or scramble sequence. Values are mean ±SEM, *p<0.05, **p<0.01, ***p<0.001.

We did not see any substantial morphological differences between the two groups in H&E staining of the aorta (Fig 5A). However, Masson-Trichrome staining showed increased deposition of collagen in the miR-181a1/b1^-/-^ mice vessels compared to the WT (All layer: WT 2.9±0.28 vs. a/b KO 3.8±0.19, p = 0.014; Vascular media: WT 5.3±0.46 vs. a/b KO 6.9±0.25, p = 0.009) (n = 5–6). (Fig 5A and 5B) The pathogenesis of the vascular stiffness in miR-181a1/b1^-/-^ mouse aorta could be due to ECM remodeling mediated by TGF-β activation as we have identified a trend to higher level of serum TGF-β in the miR-181a1/b1^-/-^ group compared to WT at 10 week (WT 117.8±5.049 ng/ml vs. a/b KO 141.1±18.13 ng/ml, p = 0.26), and a significant difference at 22 weeks of age (WT 96.81±12.58 ng/ml vs. a/b KO 139.1±10.14 ng/ml, p = 0.026). (Fig 6A) Western blot of the aorta for p-SMAD2/3, a downstream target of TFG-β, at 22 weeks showed significantly higher pSMAD2/3 in miR-181a1/b1^-/-^ aorta compared to WT (WT: 1.0±0.12 vs. a/b KO: 1.9±0.19, p = 0.006). (Fig 6B) Immunohistochemistry for pSMAD2/3 also showed higher TGF- β signaling in the miR-181a1/b1^-/-^ aorta compared to WT (Fig 6C and 6D). We have identified most of the p-SMAD2/3 staining in the vascular media (WT: 1.8±0.28% vs. a/b KO: 3.2±0.53%, p = 0.040). (Fig 6C and 6D), suggesting the potential role of VSMCs in TGF-β induced ECM remodeling.

### Activation of TGF- β pathways in VSMCs in miR-181a/b deficient mice

The thoracic aorta was excised from 12 week old mice from both genotypes (n = 4), and VSMCs and VECs were cultured into two different plates from the same aorta. Western blot data suggest a relatively pure population of VSMCs (α-Actin) and VECs (VE-Cadherin) in the corresponding cultures (Fig 6E). After passage 2 (p2), the cell-culture media was collected (without any growth factor), and TGF-β release was measured in both VSMCs and VECs. Fig 6F shows a significant increase in TGF-β release from the VSMCs of miR-181a1/b1^-/-^ mice (WT: 14.47±1.99 ng/ml vs. a/b KO: 30.57±4.51 ng/ml, p = 0.031); whereas, no significant difference was observed in the VECs between the two groups (WT: 32.30±11.00 ng/ml vs. a/b KO: 26.60±8.11 ng/ml, p = 0.70). The effect of Angiotensin II on TGF-β expression was tested in the VSMCs obtained from miR-181a1/b1^-/-^ mice and WT mice. There was an increased level of TGF-β in the miR-181a1/b1^-/-^ VSMCs at baseline (WT: 128.9±11.46 vs. a/b KO: 220.4±10.76, p = 0.001). (Fig 6G) Angiotensin II (1 μM) treatment for 48 hours in these cells showed significantly higher TGF-β in the miR-181a1/b1^-/-^ VSMCs cells (WT: 163.2±8.68 vs. a/b KO: 245.7±12.13, p<0.001) and a significant increase in TGF-β with Angiotensin II treatment in the WT VSMCs (without Ang II: 128.9±11.46 vs. Ang II: 163.2±8.68, p = 0.043). However, there was no significant increase in TGF-β expression in the miR-181a1/b1^-/-^ VSMCs cells after treatment with angiotensin II (without Ang II: 220.4±10.76 vs. Ang II: 245.7±12.13, p = 0.21). (Fig 6G) To evaluate how miR-181b affects TGF-β signaling, post transfection luciferase activity of TGF-βi was performed. Using both TargetScan and DIANA-microT, we have predicted that position 71–77 of TGF-βi 3'-UTR can be a target of miR-181b (Fig 6H, upper panel). We then performed mutagenesis at positions 72, 74, 76 and 77 from “A to C”, “A to C”, “G to C”, and “U to A”, respectively (Fig 6H, lower panel). Rat aortic vascular smooth muscle cells (A7r5) were co-transfected with TGF-βi or mutated TGF-βi reporter construct, along with miR-181b or scramble sequence. As shown in Fig 6I, 48 hr post transfection luciferase activity in TGF-βi is significantly less compared to the scramble transfected group (miR-181b: 0.048±0.004 vs. Scramble: 0.068±0.004, p = 0.005). However, mutant TGF-βi showed no difference in luciferase activity (miR-181bMUT: 0.064±0.007 vs. Scramble: 0.068±0.004, p = 0.66). (Fig 6I, Fig A in S1 File) Further, to see whether TGF-β receptor inhibition could alter the release of TGF-β through a feedback system, the WT VSMCs and miR-181a1/b1^-/-^ VSMCs cells were treated with 5μM of D4476 for 48 hours. Although there was a trend towards decreased TGF-β release in the miR-181a1/b1^-/-^ VSMCs, there were no significant changes in the release of TGF-β in both cell lines (a/b KO: not treated 220.4±10.8 vs. treated 190.1±9.7, p = 0.08; WT: not treated 128.9±11.5 vs. treated 125.7±3.6, p = 0.74). (Fig B in S1 File)

### miR-181a/b is not associated with circulating levels of Angiotensin II and expression of Angiotensin II receptors

**Fig 7:**
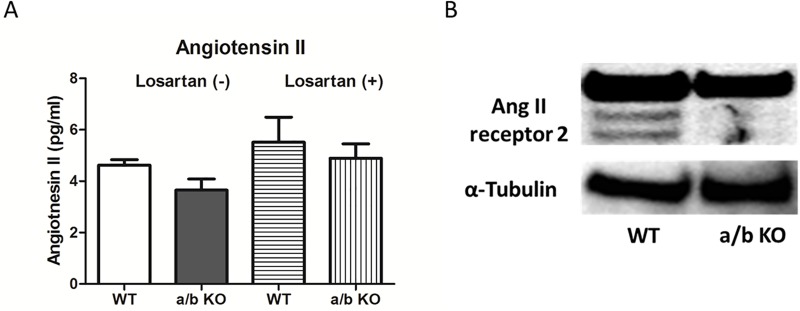
**A, Circulating levels of angiotensin II in the serum of miR-181a1/b1^-/-^ (a/b KO) and control (WT).** (n = 4) **B**, Western blot of angiotensin II receptor 2 in the aorta of miR-181a1/b1^-/-^ (a/b KO) and control (WT).

The Angiotensin II levels in the serum of the mice were tested by ELISA. (Fig 7A) There were no significant differences in Angiotensin II in the WT compared to the miR-181a1/b1^-/-^ group. (WT: 4.62±0.22vs. a/b KO: 3.65±0.43, p = 0.09). (Fig 7A) Further, although there was a trend towards higher Angiotensin II in those mice treated with losartan, there were no significant differences between the WT and the miR-181a1/b1^-/-^ group treated with losartan. (WT: 5.51±0.97 vs. a/b KO: 4.89±0.55, p = 0.60). (Fig 7A) Western blot of Angiotensin II receptor in the aortic vessels were also similar between the two groups. (Fig 7B)

### Losartan mitigates age-associated increase in BP and PWV in miR-181a/b deficient mice

**Fig 8:**
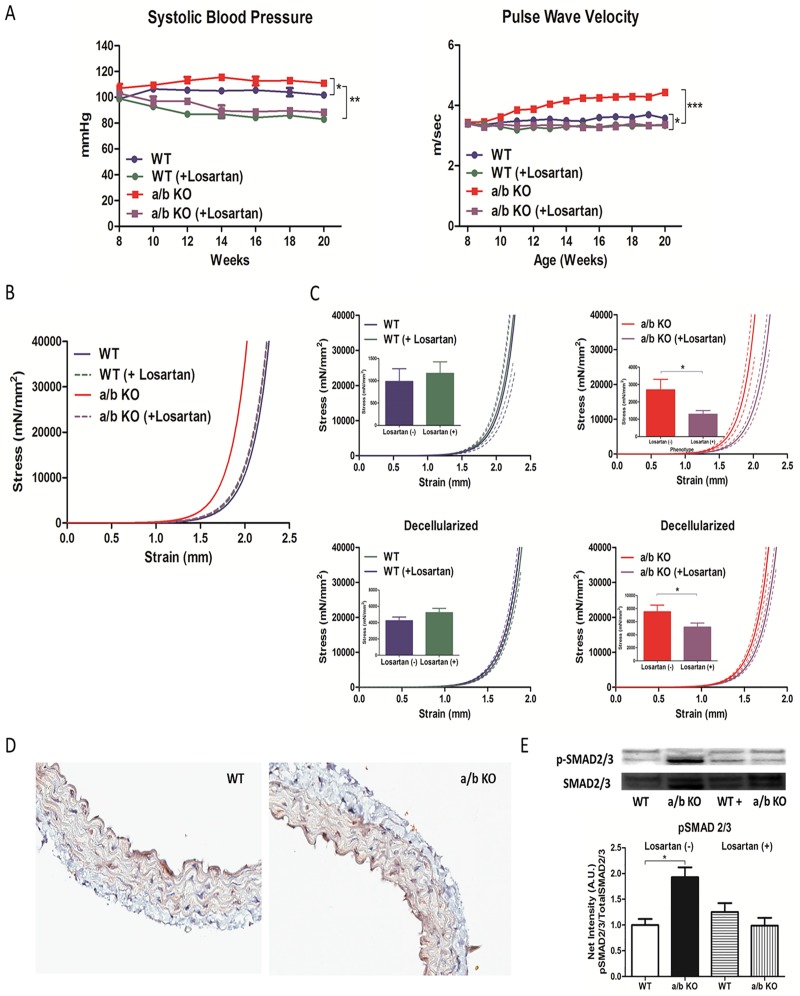
**A, Bi-weekly systolic blood pressure and weekly PWV of miR-181a1/b1^-/-^ (a/b KO) and control (WT) mice, treated and not treated with losartan (0.6g/L).** (n = 8–12) Tensile testing of the thoracic aorta from miR-181a1/b1^-/-^ (a/b KO) and control (WT) mice treated and not treated with losartan. **B**,**C** Tensile testing of control (WT) mice treated (n = 14) and not treated with losartan (n = 11), miR-181a1/b1^-/-^ (a/b KO) mice treated (n = 11) and not treated with losartan (n = 10), decellularized aortic rings from control (WT) mice treated (n = 10) and not treated with losartan (n = 8), and decellularized aortic rings from miR-181a1/b1^-/-^ (a/b KO) mice treated (n = 13) and not treated with losartan (n = 9). The bar graph shows the strength of the vessel at 1.5mm in which the bar represents mean and the error bar represent SEM. **D**, Immunohistochemistry of p-SMAD2/3 staining of the aorta treated with losartan in miR-181a1/b1^-/-^ (a/b KO) and control (WT) mice. **E**, Western blot of p-SMAD2/3 compared to total SMAD2/3 in the aorta from miR-181a1/b1^-/-^ (a/b KO) and control (WT) mice treated and not treated with losartan (n = 5). Values are mean ±SEM, *p<0.05, **p<0.01, ***p<0.001.

WT and a/b KO mice were treated with losartan (0.6 g/L) in drinking water starting at 8 weeks of age. Losartan significantly lowered the BP and PWVs (Fig 8A) in a/b KO mice compared to untreated age-matched mice from 10 weeks until 20 weeks of age. Mechanical modulus of the aorta as assessed by tensile testing also showed significant attenuation in stiffness in losartan treated miR-181a1/b1^-/-^ mice (Fig 8B). Interestingly, WT, WT + Losartan, and a/b KO + Losartan, showed identical mechanical properties and PWV profiles (Fig 8B and 8C). This further suggests that depletion of miR-181a/b promotes vascular stiffness via ECM remodeling by activating TGF-β signaling in VSMCs. Both IHC (Fig 8D) and western blot (Fig 8E) at 22 weeks showed reversal of upregulated p-SMAD2/3 signaling in miR-181a1/b1^-/-^ aorta with losartan treatment. To test whether Losartan has any direct effect on miR-181b expression in the aortic VSMCs, relative miR-181b expression was evaluated in VSMCs treated or not treated with Losartan (10 μM) for 48 hours. No significant alteration in either group was observed (No treatment: 1.16±0.17 vs. Losartan: 1.02 ± 0.09, p = 0.50). (Fig C in S1 File)

## Discussion

Hypertension is one of the major health problems of our aging population.[26, 27] Vascular stiffness is an important component of systolic hypertension and treatment to modulate vascular stiffness is suggested, as PWV has become a powerful predictor of cardiovascular events.[4, 26, 28, 29] Over the past few years, there have been numerous studies that have pointed to a major role of miRNA in hypertensive heart disease.[30–32] For example, patients with coronary artery disease have reduced circulating levels of miR-181b, suggesting the potential role of miR-181b in the pathogenesis of arterial inflammation.[7, 8] Accumulating evidence indicates that miR-181 modulates vascular function by targeting multiple key cell signaling pathways, including NF-κB signaling in the vascular endothelium as well as the PI-kinase pathway. [7, 8, 10, 33] However, there is no evidence showing the role of miR-181 in baseline vascular function. We used miR-181a1/b1-deficient mice to study this phenomenon, and have identified a phenotype associated with vascular stiffening due to activation of the TGF-β signaling cascade.[10] (Fig 2A)

Chronic down-regulation of miR-181b expression with age was associated with activation of TGF-β signaling in the VSMCs (Fig 6D, 6E, 6F, 6G and 6H). Excessive TGF-β production from VSMCs has been shown to modulate ECM growth and function.[32, 34, 35] Activated TGF-β is found to be increased in aged aortas and contributes to elastic artery stiffening with age.[36, 37] TGF-β can initiate multiple effects in the vessels including phenotypic modulation of the VECs and VSMCs. It is also associated with induction of gene expression such as collagen I and III, stimulating production of ECM. Furthermore, it also stimulates plasminogen activator-inhibitor production, which inhibits breakdown of ECM, resulting in increased ECM.[38] These cellular signaling mechanisms contribute to vascular remodeling as well as primary and secondary forms of hypertension in atherosclerosis. [38] A role for collagen deposition in the adventia of the aortic vessels has also been suggested to cause vascular stiffness potentially leading to systolic hypertension.[39]

We have also demonstrated in this study that TGF-β can be the direct target of miR-181b in the VSMCs, where miR-181b binds directly to the 3'-UTR of TGF-βi. (Fig 6H) TGF-βi (transforming growth factor, beta induced) is one of the most important components of the TGF-β signaling pathway, and it is highly abundant in the cardiovascular system. This gene encodes an RGD-containing protein that binds to type I, II and IV collagens. Thus, this protein plays a role in cell-collagen interactions (NM_009369.4). In theory, the entire miR-181 family can bind to the same 3'-UTR region of TGF-β ligand. Among the six mature family members, miR-181 a1, a2, b1, b2, c, and d, it has been found that miR-181a1 and miR-181b1 are the most prevalent in the aorta of mice.[7, 40, 41] The mature sequence of miR-181a1 and miR-181a2, and miR-181b1 and miR-181b2 are identical, but they are encoded from two different genomic loci: the miR-181a1 and miR-181b1 cluster is located on chromosome 1, and the miR-181a2 and miR-181b2 cluster is located on chromosome 9.[42] To test the baseline vascular function of miR-181 family *in vivo*, we used mice deficient for the miR-181a1-miR-181b1 cluster.[10] The miR-181a1-miR-181b1 cluster is mainly expressed in blood vessels, and both may contribute to vascular stiffness by regulating TGF-β signaling in the VSMCs. Intriguingly, VECs of the miR-181a1/b1^-/-^ mice were associated with increased eNOS activity (Fig 4), which likely blunts some effects of increased BP and vascular stiffness (Figs 2 and 4). This phenomenon might be explained by the proposed effect of TGF-β on eNOS activity reported previously. Saura, et al.[43] reported an alternative function of TGF-β which induces eNOS expression mediated via SMAD2. Further, Heger et al.[44] reported a role for TGF-β in eNOS phosphorylation and increased NO release independent of SMAD activation. It is also possible that the increase in NOS may actually be uncoupled NOS, which may be deterious to endothelial function and may also stimulate atherosclerosis. [45] There are several phosphorylation sites on eNOS. The majority of work has focused however on 2 residues, serine 1177 (Ser 1177) and threonine 495 (Thr 495). [46] It has been well documented that Akt specifically induces phosphorylation of Ser 1177 and that increases NO production. On the other hand, phosphorylation of Thr 495 has been reported to downregulate the generation of NO. Since we measured phospho-eNOS at serine 1177, it is likely that the increase in phospho-eNOS 1177 leads to an increase rather than a decrease in NO production, and not to uncoupling, reduced NO, and increased reactive oxygen species production. Furthermore, the myography experiment did not show any endothelial dysfunction nor did we find any noticeable atherosclerotic changes in the a/b KO vascular ring. Several studies have pointed out the important role of miR-181b in VECs. [7, 47–49] It has been validated that miR-181b regulates NFκB signaling by targeting importin-α3. [7, 47] Thus loss of miR-181b can activate NFκB signaling in VECs. Several studies has pointed out that NF-κB is a negative regulator of eNOS expression in VECs. [50, 51] Systemic administration of miR-181b reduced downstream NF-κB signaling by targeting importin-α3 in the vascular endothelium. [47] Recently, miR-181b has been shown to target caspase recruitment domain family member 10 (Card10) in ECs to prevent thrombin-mediated endothelial activation and arterial thrombosis.[49] This could explain why we have noticed higher p-eNOS in our miR-181a1b1^-/-^ mice. As the role of miR-181b in VECs is a very well studied topic, and vascular stiffness is mainly due to functional changes of VSMCs, we focused our study on the underlying mechanism involving miR-181b in VSMCs rather than VECs.

Losartan, an angiotensin II-type 1 receptor blocker and a known TGF-β neutralizer, is a very effective anti-hypertension medication.[23] Our results showed that treatment with losartan (0.6 g/L), which not only lowers the BP, but neutralizes TGF-β signaling, was able to mimic the role of miR-181a1/b1 during the entire time span of the experiment (11 weeks to 20 weeks), and reversed the vascular stiffness phenotype of miR-181a1/b1^-/-^ mice.[23, 52, 53] (Fig 8) Interestingly, our result showed no difference in the Angiotensin II levels in the serum of the WT and the miR-181a1/b1^-/-^ mice. This indicates that miR-181b is associated with direct inhibition of TGF-β signaling independent of Angiotensin II. Several studies have suggested that TGF-β induces miR-181 expression.[54–57] However, our result have also shown that miR-181b can directly target the 3’-UTR of TGF-βi (Fig 6H and 6I). This suggests that miR-181b may have a role in a feedback loop of TGF-β signaling. Further, there were no significant alterations in TGF-β release after inhibition of TGF-β receptor with D4476 in the VSMCs. This may suggest that the role of miR-181b in the feedback loop of the TGF-β cascade is upstream of the TGF-β receptor. Although the vascular stiffness phenotype was reversed following treatment with the TGF-β neutralizer, losartan,(2, 9, 27) losartan did not alter miR-181b expression in the VSMCs, suggesting that reversal of vascular stiffness by losartan was due to inhibition of TGF-β signaling downstream of miR-181b. Although its mechanism remains to be fully elucidated, usage of Losartan has been reported to be associated with reduced expression of TGFβ ligands, receptors, and activators. (13) These results may suggest that the main role of losartan in the reversal of the phenotype was not only due to reduced expression of TGFβ ligands which may not have been significant as we did not see any changes in the expression of miRNA-181b, but by downstream inhibition of TFG-β targets.

Hypertension and increased vascular stiffness are common with aging.[28, 58] Reducing vascular stiffness could be an important adjunct to controlling blood pressure to reduce the risk of cardiovascular events in the elderly. We have identified the potential role of miR-181a1/b1 in the development of vascular stiffness, which ultimately was associated with higher systolic blood pressure via activation of TGF-β. Even though losartan is currently in widespread clinical use for the management of hypertension and stroke, miR-181b could potentially be a new or additive therapeutic target for intervention, targeting the TGF-β cascade independent of Angiotensin II.

## Conclusion

More than 500 miRNAs have been identified in humans, with few of them being involved in regulating smooth muscle cell phenotype, and modulating inflammatory response in endothelial cells and macrophages in the process of arterial remodeling and atherosclerosis.[59] The present study shows a role of miR-181b in regulating TGF-β signaling, in the smooth muscle cells, which modulates vascular stiffness. Increased TGF-β expression is observed in aged aorta, which is associated with increased extracellular matrix deposition, increased cross-linking and ultimately vascular remodeling.[38, 60–62] Not only is lowering blood pressure an important target in clinical practice today, but central vascular stiffness as determined by pulse wave velocity, has become both a powerful predictor of cardiovascular events, and an important parameter for targeting therapy.[26] miRNA181b could be a potential therapeutic target in modulating vascular stiffness along with the treatment of hypertension.

## Supporting information

S1 File **Fig A. Transfection efficiency of rat aortic smooth muscle cells.** Cell image represent transfection efficiency of A7r5 cell lines using electroporation. Red color represents mCherry tagged beta-actin. Green color represents FITC tagged at the 3'-end of miR-181b. **Fig B. Effect of TGF-**β**1 inhibitor (D4476) on vascular smooth muscle cells.** TGF-β release in the WT VSMCs and miR-181a1/b1^-/-^ VSMCs cells treated with 5μM of D4476 for 48 hours. (n = 4–8) Values are mean ±SEM, *p<0.05, **p<0.01, ***p<0.001. **Fig C. Effect of losartan on miR-181b expression.** miR-181b expression after losartan treatment (10u<M for 48hrs) in the aortic VSMCs. Values are mean ±SEM, *p<0.05, **p<0.01, ***p<0.001.
(DOCX)

S2 File Raw data. (ZIP)
