# Supplementary figures and images for: miR-181b regulates vascular stiffness age dependently in part by regulating TGF-β signaling
Source: PLoS One. 2017 Mar 21;12(3):e0174108. doi: 10.1371/journal.pone.0174108 (PMC5360327; doi:10.1371/journal.pone.0174108)

Supporting Document

**Supplement A**


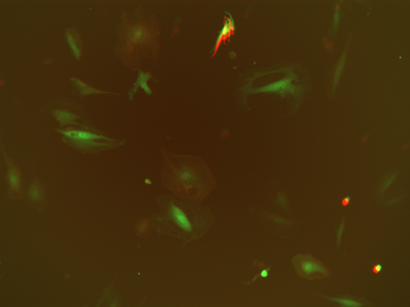


**Supplement B**


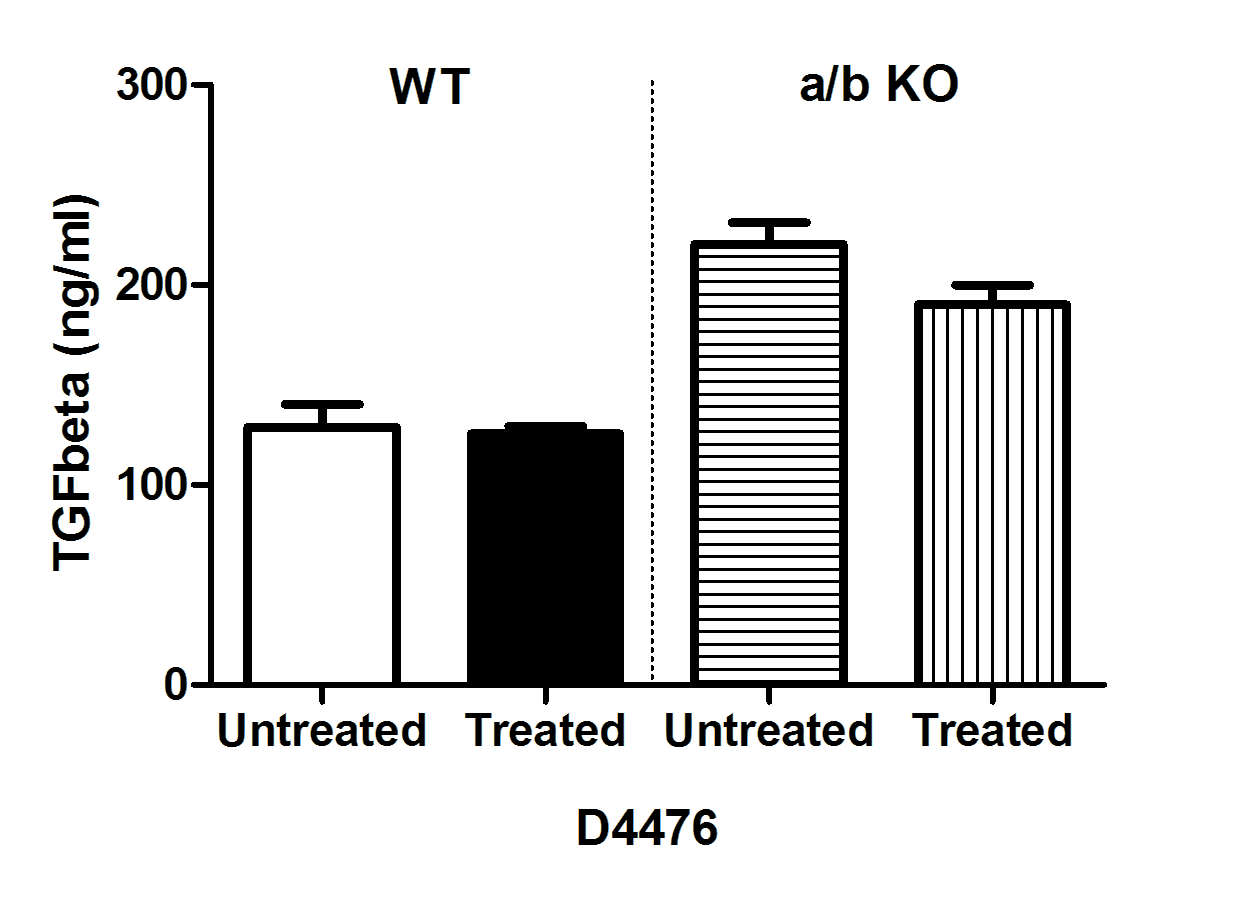


**Supplement C**


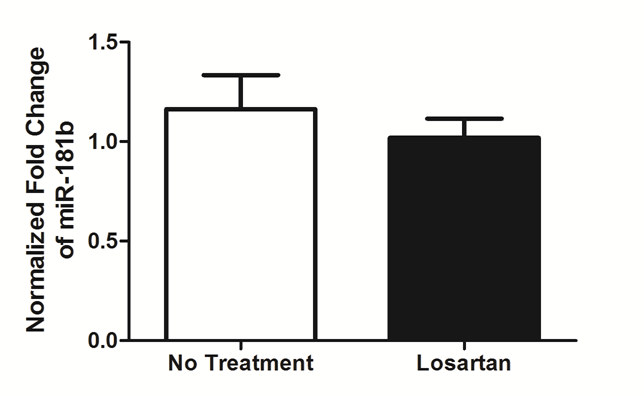

Supplement: S1 File — Fig A. Transfection efficiency of rat aortic smooth muscle cells. Cell image represent transfection efficiency of A7r5 cell lines using electroporation. Red color represents mCherry tagged beta-actin. Green color represents FITC tagged at the 3'-end of miR-181b. Fig B. Effect of TGF-β1 inhibitor (D4476) on vascular smooth muscle cells. TGF-β release in the WT VSMCs and miR-181a1/b1-/- VSMCs cells treated with 5μM of D4476 for 48 hours. (n = 4–8) Values are mean ±SEM, *p<0.05, **p<0.01, ***p<0.001. Fig C. Effect of losartan on miR-181b expression. miR-181b expression after losartan treatment (10u<M for 48hrs) in the aortic VSMCs. Values are mean ±SEM, *p<0.05, **p<0.01, ***p<0.001. (DOCX) [file pone.0174108.s001.docx]
